# Gestationsdiabetes (GDM) (Update 2023)

**DOI:** 10.1007/s00508-023-02181-9

**Published:** 2023-04-20

**Authors:** Alexandra Kautzky-Willer, Yvonne Winhofer, Herbert Kiss, Veronica Falcone, Angelika Berger, Monika Lechleitner, Raimund Weitgasser, Jürgen Harreiter

**Affiliations:** 1 Gender Medicine Unit, Abteilung für Endokrinologie und Stoffwechsel, Universitätsklinik für Innere Medizin III, Medizinische Universität Wien, Währinger Gürtel 18–20, 1090 Wien, Österreich; 2 Abteilung für Geburtshilfe und feto-maternale Medizin, Universitätsklinik für Frauenheilkunde, Medizinische Universität Wien, Wien, Österreich; 3 Abteilung für Neonatologie, Pädiatrische Intensivmedizin und Neuropädiatrie, Universitätsklinik für Kinder- und Jugendheilkunde, Medizinische Universität Wien, Wien, Österreich; 4 Interne Abteilung, Landeskrankenhaus Hochzirl – Natters, Hochzirl, Österreich; 5 Abteilung für Innere Medizin/Diabetologie, Privatklinik Wehrle-Diakonissen, Salzburg, Österreich

**Keywords:** Gestationsdiabetes, Diabetische Fetopathie, Prädiabetes, Typ 2 Diabetes mellitus, Übergewicht/Adipositas, Kardiovaskuläres Risiko, Schwangerschaft, Schwangerschaftskomplikationen, Geburtshilfliche Versorgung, Neonatale Versorgung, Gestational diabetes mellitus, Diabetic fetopathy, Prediabetes, Type 2 diabetes mellitus, Overweight/obesity, Cardiovascular risk Pregnancy, Pregnancy complications, Neonatal care

## Abstract

Gestationsdiabetes (GDM) wird als Glukosetoleranzstörung definiert, die erstmals in der Schwangerschaft festgestellt wird. GDM ist mit einer erhöhten feto-maternalen Morbidität sowie Langzeitkomplikationen bei Mutter und Kind assoziiert. Frauen, die die Kriterien eines manifesten Diabetes mellitus bereits in der Frühschwangerschaft erfüllen (Nüchternplasmaglukose ≥ 126 mg/dl, Spontanglukosemessung ≥ 200 mg/dl oder HbA_1c_ ≥ 6,5 % vor der 20. Schwangerschaftswoche) sollen als Schwangere mit manifestem Diabetes klassifiziert und ebenso behandelt werden. Ein Screening auf unerkannten Typ 2 Diabetes bei der ersten pränatalen Kontrolle wird besonders bei Frauen mit hohem Risiko (Anamnese eines GDM/Prädiabetes, Fehlbildungen, Totgeburt, wiederholte Aborte oder Geburtsgewicht über 4500 g in früheren Schwangerschaften, Adipositas, metabolisches Syndrom, Alter > 35 Jahre, bei Gefäßerkrankungen, Auftreten von Diabetessymptomen wie Glukosurie, ethnische Zugehörigkeit zu Gruppen mit hohem Risiko [arabisch, S und SO-asiatisch, lateinamerikanisch]) empfohlen. GDM wird durch einen oralen Glukosetoleranztest (oGTT, 120 min; 75 g Glukose) oder durch Nüchternplasmaglukose ≥ 92 mg/dl diagnostiziert. Bei hohem Risiko kann ein oGTT bereits im ersten Trimenon sinnvoll sein, zwischen der 24.–28. Schwangerschaftswoche muss dieser Test aber in jedem Fall bei allen Schwangeren mit bis dahin unauffälligen Glukosewerten im Rahmen der Mutter-Kind-Pass-Untersuchung durchgeführt werden. Nach WHO Empfehlungen basierend auf der „Hyperglycemia and Adverse Pregnancy Outcome (HAPO) study“ liegt ein GDM vor, wenn die Plasmaglukose nüchtern 92 mg/dl, nach 60 min 180 mg/dl oder nach 120 min 153 mg/dl überschreitet (Internationale Konsensuskriterien). Ein einziger erhöhter Wert ist für die Diagnose ausreichend und bedarf bereits einer strikten Stoffwechselkontrolle. Nach bariatrischer Operation wird aufgrund der Gefahr einer postprandialen Hypoglykämie die Durchführung eines oGTT nicht empfohlen. Alle Frauen mit GDM müssen eine Ernährungsberatung erhalten und ihre Blutzuckerwerte (4 Messzeitpunkte) regelmäßig kontrollieren. Ebenso sollte, falls nicht kontraindiziert, die körperliche Aktivität erhöht werden. Falls die Blutzuckerspiegel nicht im Therapiezielbereich liegen (nüchtern < 95 mg/dl und 1 h postprandial < 140 mg/dl, Evidenzklasse B) soll als erste Wahl eine Insulintherapie initiiert werden (Evidenzklasse A). Neben der mütterlichen Stoffwechselüberwachung sind geburtshilfliche Kontrollen und ein ultraschallgestütztes, fetales Monitoring notwendig, um die mütterliche und fetale/neonatale Morbidität und die perinatale Mortalität möglichst gering zu halten (Evidenzklasse A). Im Rahmen der neonatalen Untersuchungen müssen bei Neugeborenen von Müttern mit GDM Blutzuckerkontrollen erfolgen und bei Erfordernis geeignete Maßnahmen eingeleitet werden. Nach der Entbindung (4–12 Wochen post partum) wird neuerlich die Durchführung eines oGTT (75 g; WHO Kriterien) bei allen Frauen mit GDM empfohlen, um eine über die Schwangerschaft hinaus bestehende Glukosetoleranzstörung auszuschließen. Bei Normalbefund sollen alle 2–3 Jahre regelmäßig weitere Testungen (Nüchternblutzucker, Spontanglukose, HbA_1c_ oder oGTT) erfolgen (Evidenzklasse B). Alle Frauen sollen über ihr deutlich erhöhtes Risiko für Typ 2 Diabetes, das höhere kardiovaskuläre Risiko, sowie über entsprechende Präventionsmaßnahmen, informiert werden. Dazu gehören Lebensstilmaßnahmen, wie Gewichtsreduktion bei Übergewicht, gesunde Ernährung und ausreichend körperliche Aktivität (Evidenzklasse A). Auch die Kinder sollen hinsichtlich einer unauffälligen Entwicklung regelmäßig nachuntersucht werden, da in rezenten Untersuchungen höheres Risiko für Übergewicht und Adipositas sowie erhöhte Glukoseparameter festgestellt wurden. Wenn möglich sollte die gesamte Familie über Lebensstilmaßnahmen zur Aufrechterhaltung/Verbesserung der Gesundheit informiert werden.

## Abstract (en)

Gestational diabetes (GDM) is defined as any degree of glucose intolerance with onset during pregnancy and is associated with increased feto-maternal morbidity as well as long-term complications in mothers and the offspring. Women detected to have diabetes early in pregnancy receive the diagnosis of overt, non-gestational, diabetes (glucose: fasting ≥ 126 mg/dl, spontaneous ≥ 200 mg/dl or HbA_1c_ ≥ 6.5% before 20 weeks of gestation). GDM is diagnosed by an oral glucose tolerance test (oGTT) or increased fasting glucose (≥ 92 mg/dl). Screening for undiagnosed type 2 diabetes at the first prenatal visit is recommended in women at increased risk (history of GDM/pre-diabetes; malformation, stillbirth, successive abortions or birth weight > 4500 g previously; obesity, metabolic syndrome, age > 35 years, vascular disease; clinical symptoms of diabetes (e.g. glucosuria) or ethnic origin with increased risk for GDM/T2DM (Arab, South- and Southeast Asian, Latin American)) using standard diagnostic criteria. Performance of the oGTT (120 min; 75 g glucose) may already be indicated in the first trimester in high-risk women but is mandatory between gestational week 24–28 in all pregnant women with previous non-pathological glucose metabolism. Following WHO recommendations, which are based on the Hyperglycemia and Adverse Pregnancy Outcome (HAPO) study, GDM is defined, if fasting venous plasma glucose is ≥ 92 mg/dl or 1 h ≥ 180 mg/dl or 2 h ≥ 153 mg/dl after glucose loading (international consensus criteria). In case of one pathological value a strict metabolic control is mandatory. After bariatric surgery we do not recommend to perform an oGTT due to risk of postprandial hypoglycemia. All women with GDM should receive nutritional counseling, be instructed in blood glucose self-monitoring and motivated to increase physical activity to moderate intensity levels—if not contraindicated (Evidence level A). If blood glucose levels cannot be maintained in the therapeutic range (fasting < 95 mg/dl and 1 h after meals < 140 mg/dl, Evidence level B) insulin therapy should be initiated as first choice (Evidence level A). Maternal and fetal monitoring is required in order to minimize maternal and fetal/neonatal morbidity and perinatal mortality. Regular obstetric examinations including ultrasound examinations are recommended (Evidence level A). Neonatal care of GDM offspring at high risk for hypoglycaemia includes blood glucose measurements after birth and if necessary appropriate intervention. Monitoring the development of the children and recommendation of healthy lifestyle are important issues to be tackled for the whole family. After delivery all women with GDM have to be reevaluated as to their glucose tolerance by a 75 g oGTT (WHO criteria) 4–12 weeks postpartum. Assessment of glucose parameters (fasting glucose, random glucose, HbA_1c_ or optimally oGTT) are recommended every 2–3 years in case of normal glucose tolerance. All women have to be instructed about their increased risk of type 2 diabetes and cardiovascular disease at follow-up. Possible preventive meassures, in particular lifestyle changes as weight management and maintenance/increase of physical activity should be discussed (evidence level A).

## Grundsatz Statement

Bei Frauen mit in der Schwangerschaft erstmals aufgetretener oder diagnostizierter Glukosetoleranzstörung besteht gegenüber Schwangeren mit normaler Glukosetoleranz ein höheres Risiko für perinatale Morbidität und Mortalität und operative Entbindungen und ein höheres Risiko nach Entbindung einen Diabetes mellitus Typ 2 (T2DM) und kardiovaskuläre Komplikationen zu entwickeln [1, 2]. Frauen mit Gestationsdiabetes (GDM) und strikter metabolischer Kontrolle haben bessere Schwangerschaftsergebnisse als Frauen mit GDM, die nicht behandelt werden [3, 4]. Antenatale Lifestyle-Interventionen (strukturierte Ernährungsempfehlungen und Bewegungsprogramme) führen zu besseren Schwangerschafts-Outcomes [5]. Patientinnen, bei denen sich während der Schwangerschaft ein Typ 1 Diabetes mellitus manifestiert, sowie solche mit einem bereits präkonzeptionell oder zu Schwangerschaftsbeginn bestehenden, aber erst in der Gravidität diagnostizierten anderen Diabetestyp, sollen wie Patientinnen mit präkonzeptionell bekanntem Diabetes mellitus behandelt und überwacht werden (siehe Leitlinie: Gravidität bei vorbestehendem Diabetes). Ein präkonzeptionell bestehender Diabetes mellitus kann angenommen werden, wenn bereits vor der 20. Schwangerschaftswoche die Kriterien für einen manifesten Diabetes mellitus erfüllt sind: Nüchternblutzucker ≥ 126 mg/dl oder Spontanmessung ≥ 200 mg/dl; bzw. 2 h-Wert im oralen Glukosetoleranztest (oGTT) ≥ 200 mg/dl oder HbA_1c_ ≥ 6,5 %. Frauen mit GDM haben postpartal ein deutlich erhöhtes Risiko für die Entwicklung eines T2DM und sollen deshalb über Präventionsmaßnahmen informiert und lebenslang überwacht werden [6].

## Risikoevaluierung und Diagnose (Abb. 1)

Bei Erstvorstellung beim Frauenarzt werden schwangere Frauen bezüglich ihres Risikos für GDM oder Diabetes mellitus eingestuft. Bei Vorliegen eines höheren Risikos (siehe unten) soll die Frau möglichst früh hinsichtlich einer Glukosestoffwechselstörung untersucht werden: Dies kann durch eine Nüchternglukosemessung, eine Spontanglukosemessung, eine HbA_1c_-Bestimmung und/oder Durchführung eines oGTT erfolgen.

**Figure 1:**
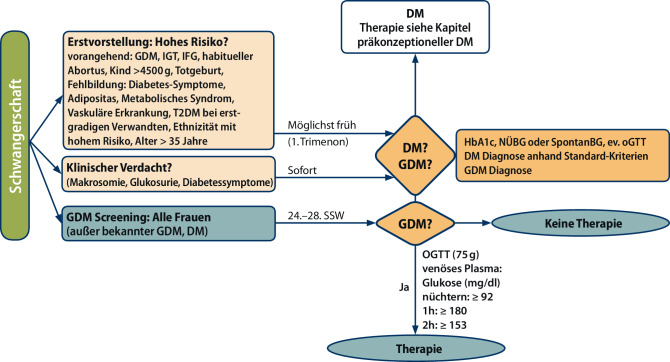

Hohes Risiko für GDM bzw. Risiko für vorbestehende, unerkannte Stoffwechselstörung (Prädiabetes oder Diabetes) besteht bei:

- GDM in einer früheren Schwangerschaft
- Prädiabetes in der Anamnese (gestörte Glukosetoleranz und/oder Nüchternglukose > 100 mg/dl)
- Kongenitale fetale Fehlbildung in einer früheren Schwangerschaft
- Geburt eines Kindes > 4500 g
- Intrauteriner Fruchttod (IUFT)
- Habitueller Abortus (> 3 Fehlgeburten hintereinander)
- Diabetes-Symptome
- Adipositas (BMI ≥ 30 kg/m^2^)
- Alter über 35 Jahre
- Metabolisches Syndrom
- Vaskuläre Erkrankung (Koronare Herzkrankheit (KHK), Insult (cAVK), periphere arterielle Verschlusskrankheit (pAVK))
- Familienanamnese von T2DM bei erstgradigen Verwandten
- Ethnizität (z. B. arabisch, S‑ und SO-asiatisch, lateinamerikanisch)

Bei Auftreten von diabetes-spezifischen Symptomen oder klinischer Auffälligkeiten (Polydipsie, Polyurie, Glukosurie; Makrosomie) ist ein Test – auch bei unauffälligem Vorbefund und unabhängig von der Schwangerschaftswoche – unmittelbar durchzuführen.

In einer multizentrischen österreichischen Studie waren ein GDM in einer früheren Schwangerschaft, das Auftreten einer Glukosurie, Übergewicht (präkonzeptioneller BMI > 27 kg/m^2^), ein Alter über 30 Jahre und der Verdacht auf Makrosomie im Ultraschall die besten unabhängigen Prädiktoren für einen GDM [7], wobei das Risiko bei vorangegangenem GDM fast 3‑fach, ansonsten ungefähr 2‑fach erhöht war. Eine multinationale europäische Studie zeigte, dass fast jede 4. adipöse Frau bereits vor der 20. Schwangerschaftswoche erhöhte Blutzuckerwerte im Sinne eines GDMs nach IADPSG/WHO 2013 Kriterien und Parameter des metabolischen Syndroms aufwies [8].

Um geburtshilfliche Komplikationen, wie fetale Malformationen, Makrosomie, IUFT, Schulterdystokie oder sekundäre Sectio zu vermeiden, ist eine Früherkennung vom GDM bereits in der Frühschwangerschaft wünschenswert. Obwohl Frauen, die einen GDM in der zweiten Hälfte der Schwangerschaft entwickeln, bereits in der Frühschwangerschaft deutlich erhöhte Nüchternblutzucker-, sowie HbA_1c_ und C‑Peptid Werte zeigen, bleibt die Analyse von derartigen Surrogaten des Glukosestoffwechsels (Nüchternblutzucker, HbA_1c_, C‑Peptid, HOMA‑B, HOMA-IR) in der Frühschwangerschaft für die Diagnose eines GDMs nur beschränkt aussagekräftig [9]. Die Erstellung von Prädiktionsmodellen, welche gleichzeitig anamnestische Faktoren und biochemische Variablen untersuchen, könnte zur Früherkennung von GDM führen und die Risiken für unerwünschte geburtshilfliche Outcomes reduzieren [10].

Alle Schwangeren erhalten im Rahmen der 2. Mutter-Kind-Pass-Labor-Vorsorge Untersuchung zwischen der 24.–28. Schwangerschaftswoche einen 75 g oGTT [11–13]. Durch die Einführung des oGTTs im Mutter-Kind-Pass konnte in Österreich eine Reduktion der IUFT Fälle bei schwangeren Frauen mit einem erhöhten Risiko für GDM erreicht werden [14]. Bei Frauen mit bereits diagnostiziertem GDM oder Diabetes bzw. wenn der unmittelbar gemessene Nüchternglukosewert (venöse Plasmaglukose) 92 mg/dl oder höher ist, sollte auf den oGTT verzichtet werden. Ausgenommen von der Durchführung eines oGTT sind auch Frauen nach bariatrischer/metabolischer Chirurgie, da das Risiko einer postprandialen Hypoglykämie (Dumping Phänomen) nach der Ingestion der Glukoselösung besonders hoch ist [15]. Nach bariatrischer Operation werden daher regelmäßige Blutzuckerselbstkontrollen zur Diagnose eines GDM herangezogen. Ebenso ist die Verwendung eines kontinuierlichen Glukosemesssystem (CGMS, FGM) in diesem Fall denkbar [15].

## Methodik: Diagnostischer 75 g oraler Glukosetoleranztest (oGTT)

Der Test soll bei allen Frauen mit bisher unauffälligen oder unbekannten Blutglukosewerten in der Schwangerschaft zwischen 24–28. Schwangerschaftswoche morgens nach mindestens achtstündiger Nahrungskarenz durchgeführt werden. Eine Änderung der bisherigen Ernährung, eine Reduktion der Kohlenhydrate oder Diäten vor dem Test sollten vermieden werden. Ebenso sollten vor dem Test keine außergewöhnlichen körperlichen Belastungen erbracht werden. Der Testbeginn sollte zwischen 6.00 h und 9.00 h erfolgen, da die Glukosetoleranz tageszeitlichen Änderungen unterliegt. Die Schwangere soll die Glukoselösung (75 g Glukose in 300 ml Wasser) innerhalb von 5 min trinken, während des Testes sitzen (liegende Position vermeiden, keine unnötige körperliche Aktivität) und nicht rauchen. Zur GDM Diagnostik sollen Blutglukosewerte ausschließlich mit einer qualitätsgesicherten Methode in venösem Plasma direkt gemessen werden oder in venösem Vollblut gemessen und mit einem Faktor von 1,11 (+ 11 %) in venöse Plasmawerte umgerechnet werden.

Um möglichst exakte oGTT Resultate zu erhalten ist es erforderlich gewisse Standards zu berücksichtigen [12]. Diese sind wie folgt (abgeleitet nach [12]):

- Messungen aus venösem Plasma und nicht aus Kapillarblut
- Messung in einem zertifizierten Labor nach zertifizierten Methoden, um präanalytische Fehler zu minimieren.
- Am Testtag ist vor dem oGTT die eine Einnahme kontrainsulinärer Medikamente (z. B. Thyroxin, Progesteron, Glukokortikoide, Sympathikomimetika) zu vermeiden.
- Nach Einleitung der fetalen Lungenreife mittels Glukokortikoiden sollte man bis zur Testdurchführung mindestens fünf Tage zuwarten.
- Bei Fieber, akuten Erkrankungen oder verordneter Bettruhe ist der Test bis zur vollständigen Genesung zu verschieben.
- Bei operativen Eingriffen am Magen-Darm-Trakt (z. B. bariatrische Operation) ist die Aussagekraft eines oGTT limitiert. Zudem besteht die Gefahr eines Dumping-Syndroms. In diesem Fall sollte eine Blutzuckerselbstmessung über mehrere Tage erfolgen und Blutzuckerprofile zur Bewertung herangezogen werden.
- Bei Hyperemesis gravidarum oder stärkerer Schwangerschaftsübelkeit ist der Test um einige Tage zu verschieben.

Die internationale Klassifikation (Tab. 1; [13, 16]) beruht auf Evidenz-basierten (= HAPO-Studie) Blutzuckergrenzwerten [17, 18]. Ab einem pathologischen Wert ist ein GDM diagnostiziert. In einer aktuellen randomisiert kontrollierten Studie, die die HAPO GDM Diagnosekriterien (0–1–2 h: 92–180–153 mg/dl) mit höheren Diagnosekriterien (0–2 h: 99–162 mg/dl) verglich und kindliche und mütterliche Schwangerschaftsoutcomes untersuchte, konnte kein signifikanter Unterschied im Primäroutcome large for gestational age Geburt beobachtet werden [19]. In Sekundäranalysen wurde eine erhöhte Inanspruchnahme medizinischer Leistungen, aufgrund häufigerer GDM Diagnosen bei niedrigeren Grenzwerten, aber auch ein signifikant niedrigeres Risiko für Präeklampsie, Gewichtszunahme und häufigeres Stillen in der Gruppe mit niedrigeren Diagnosekriterien festgestellt.

**Table 1:** 

Zeitpunkt	Venöses Plasma (mg/dl)
Nüchtern	≥ 92
1 h	≥ 180
2 h	≥ 153

*oGTT* oraler Glukosetoleranztest, *WHO* World Health Organization, *IADPSG* International Association of the Diabetes and Pregnancy Study Groups

Anhand von Auswertungen der Schwangerschaftsergebnisse an fünf österreichischen Zentren konnte auch ein einfacher Algorithmus und Risiko-Score zur Vorhersage des GDM entwickelt werden, der auf der Messung der Nüchternplasmaglukose beruht und im Einzelfall herangezogen werden kann [21].

## Prävention

Die Prävention von GDM wurde in zahlreichen Studien untersucht und dabei an verschiedenen Risikogruppen getestet. Bei Frauen mit Adipositas konnte in den bisherigen großen Studien mit Lebensstilintervention keine Verbesserung im fetalen Outcome (large for gestational age, LGA) oder eine Verbesserung der mütterlichen Stoffwechselsituation oder GDM Prävalenz erreicht werden [22, 23]. In der DALI Studie konnte im Vergleich drei verschiedener Interventionsgruppen (gesunde Ernährung, körperliche Aktivität, Kombination aus beidem) eine signifikante Gewichtsabnahme in der kombinierten Interventionsgruppe im Vergleich zur Kontrolle gezeigt werden, dies hatte aber keinen Einfluss auf die mütterlichen oder kindlichen Outcomes bei Geburt [22, 23]. Bei adipösen Frauen konnte mit Ernährungsmaßnahmen das GDM Risiko, sowie eine kindliche Makrosomie verringert werden. Dies konnte bei körperlicher Aktivität nicht beobachtet werden. Vitamin D Supplementation in der Schwangerschaft führte zwar zu einer signifikanten Reduktion der Nüchternglukose im 3. Trimenon, dies hatte aber klinisch keine Relevanz und konnte die GDM Prävalenz bzw. das Risiko für LGA-Geburten bei adipösen, schwangeren Frauen im Vergleich zu Placebo trotz suffizienter Vitamin D‑Werte nicht reduzieren [24]. Trotz der signifikanten Erhöhung von Vitamin D und einer hohen Suffizienzrate waren durch eine Vitamin D Supplementation in der Schwangerschaft im Vergleich zu Placebogruppe auch die Lipidparameter in den Behandlungsgruppen nicht signifikant unterschiedlich [25]. Eine Supplementation mit Probiotika und Myoinositol konnte das GDM Risiko verringern. Bei adipösen schwangeren Frauen konnte unter Gabe von Metformin keine Reduktion des GDM Risikos und keine Verbesserung des mütterlichen Stoffwechsels oder Geburtsoutcomes erreicht werden [22]. Die bisherigen Studien zeigen, dass der Beginn einer Lebensstilmodifikation vor dem ersten Trimester oder idealerweise bereits bei Kinderwunsch sinnvoll ist. [5, 22]. Eine rezente Metaanalyse zeigt, dass die Implementierung strukturierter antenataler Interventionsprogramme (Ernährung, Bewegung oder beides kombiniert) das Auftreten eines Gestationsdiabetes vermindern und die mütterlichen Schwangerschafts-Outcomes verbessern können, die neonatalen Ergebnisse aber nur durch Diät beeinflusst werden konnten [5].

## Therapie (Abb. 2)

### Diabetologische/Internistische Betreuung

Erstellung eines individuellen Therapieplans bestehend aus einer Lebensstilmodifikation mit Ernährung, Bewegung und Blutglukoseselbstmessungen:

- Ernährung: Je nach Körpergewicht und körperlicher Aktivität ausgerichteter Diätplan (bei Normalgewicht ca. 24–30 kcal/kg: 40–50 % Kohlenhydrate, 30–35 % Fett und 20 % Eiweiß). Auf schnell resorbierbare Kohlenhydrate sollte verzichtet werden. Eine ballaststoffreiche Ernährung (ca. 30 g/Tag) ist zu empfehlen. Die ausreichende Versorgung mit Mineralstoffen und Vitaminen ist zu berücksichtigen (Eisen, Folsäure, Vitamin D, Kalzium, Vitamin B, Magnesium, Jod). Hier gelten die nationalen Referenzwertempfehlungen für Nahrungszufuhr in der Schwangerschaft (D-A-CH Referenzwerte, www.oege.at) Die täglich empfohlene Proteinzufuhr in der Schwangerschaft entspricht der einer gesunden Schwangerschaft (60–80 g/Tag). Eine Aufteilung der Mahlzeiten auf 3 kleine bis mittlere Hauptmahlzeiten und 2–4 kleine Zwischenmahlzeiten inklusive Abendsnack sollte erfolgen [26]. Für eine DASH- oder Mediterrane Ernährung werden sowohl präventive als auch outcome-orientiert positive Daten bezüglich GDM berichtet [27]. Die Endocrine Society empfiehlt bei Adipositas eine Kalorienrestriktion um etwa ein Drittel, so keine deutliche Gewichtsreduktion (bis maximal 5 kg) und Katabolismus auftritt. Die minimale Aufnahme liegt zwischen 1500 und 2000 kcal/Tag [27]. Bei einer Low Carb-Ernährung mit nur 35–40 % Kalorien aus Kohlenhydraten wird eine ausgleichende Protein- und Fettzufuhr vorwiegend pflanzlichen Ursprungs empfohlen. In einer Sekundäranalyse einer GDM Präventionsstudie konnte in der Ernährungsinterventionsgruppe mit reduzierter Kohlenhydratzufuhr zwar eine niedrigere Gewichtszunahme in der Schwangerschaft aber auch ein Zusammenhang mit vermutlich Lipolyse induzierten, höheren Nüchternglukosewerten, freien Fettsäuren und Ketonkörpern beobachtet werden [28]. Auch im Nabelschnurblut konnten erhöhte freie Fettsäuren festgestellt werden, wobei Langzeitbeobachtungen leider fehlen. Eine Gewichtskontrolle sollte bei jedem Kontrollbesuch erfolgen bzw. selbstständig wöchentlich von der Patientin dokumentiert werden.
  Die Gewichtszunahme in der Schwangerschaft sollte dabei den Empfehlungen des Institute of Medicine folgen (Tab. 2; [29]).

**Figure 2:**
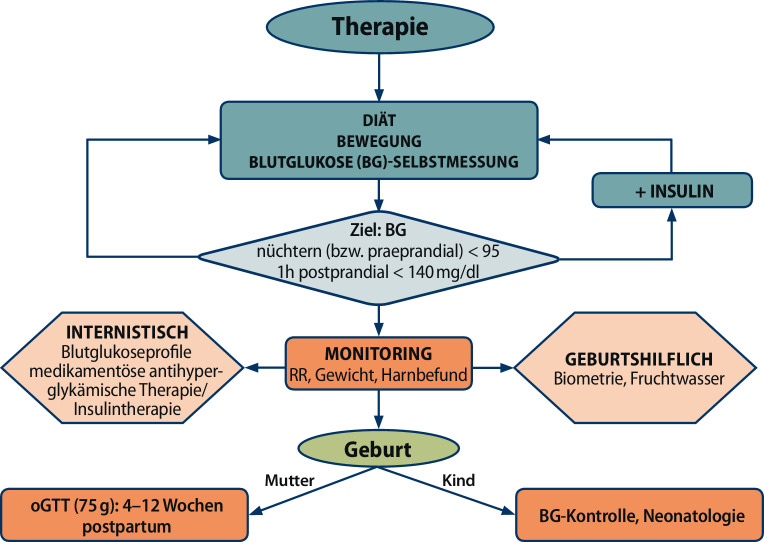
- Schulung in Blutzucker-Selbstmessung. Dokumentation der Glukose-Profile: mindestens 4 Messungen täglich (nüchtern, 1 h postprandial). Bei Beginn einer Insulintherapie sollte die Patientin über die Symptome und Risiko von Hypoglykämien, sowie über das richtige Verhalten in dieser Situation geschult werden. Eine schlechte Adhärenz zu regelmäßigen selbstständigen Blutzuckermessungen steht mit einem erhöhten Präeklampsierisiko in Zusammenhang und war in einer Studie mit Zugehörigkeit zu niedriger sozialer Klasse, nichteuropäischer Herkunft und Diabetes in der Familienanamnese assoziiert [30]. Hingegen konnten bei guter Adhärenz mit guter Blutzuckereinstellung keine Unterschiede zwischen täglich 4 Messungen und Messungen jeden zweiten Tag in mütterlichen und kindlichen Outcomes festgestellt werden [31]. Eine Reduktion der Blutzuckermessungen (Messung alle 2 Tage) kann bei guter Blutzuckereinstellung und fortgeschrittener Schwangerschaft überlegt werden. Sollte eine kontinuierliche Glukosemessung erfolgen sind folgende Blutzuckerwerte anzustreben: Zielbereich 63–140 mg/dl, Zeit im Zielbereich (Time in Range) > 70 %, Zeit unter Zielbereich < 4 %, Zeit über Zielbereich < 25 % [11]
- Bewegung: Bei einer unproblematischen Schwangerschaft ist regelmäßige moderate körperliche Aktivität ein weiterer Bestandteil des Therapiekonzepts. Die Aktivitätszeit sollte dabei mindestens 150 min pro Woche betragen und sollte in den Alltag integriert werden. Bei Ausübung von Sport sollten Sportarten gewählt werden, die mit einer Schwangerschaft vereinbar sind (kein Kontaktsport, Kampfsport, Sportarten mit hoher Sturz- oder Verletzungsgefahr) und dem jeweiligen Trainingszustand entsprechen. Zur GDM-Prävention mittels körperlicher Aktivität vor der Schwangerschaft oder in der Frühschwangerschaft und adäquater oben beschriebener Ernährung (DASH-, mediterrane Ernährung) liegt eine Metaanalyse aus 40 Studien vor [32].
- Therapieziele und pharmakologische Therapie:
  Bei unzureichender Einstellung durch Lebensstilmaßnahmen ist unmittelbar eine medikamentöse Therapie einzuleiten. Insulin sollte gegenüber oralen glukosesenkenden Medikamenten aufgrund der deutlich besseren Studienlage und keiner Plazentagängigkeit bevorzugt eingesetzt werden [11].

**Table 2:** 

BMI	BMI-Limits (kg/m^2^) (WHO)	Empfohlene Zunahme während des SS (kg)	Empfohlene Gewichtszunahme/Woche (kg/Woche) (2. + 3. Trimenon)
Untergewicht	< 18,5	13–18	0,51
Normalgewicht	18,5–24,9	11–16	0,42
Übergewicht	25,0–29,9	7–11	0,28
Adipositas	≥ 30,0	5–9	0,22

*BMI* Body Mass Index,*WHO* World Health Organization

Werden die Grenzwerte zu einem Messzeitpunkt zu 50 % überschritten (Tab. 3), ist eine individuell anzupassende Insulintherapie, zu beginnen. Liegen Nüchternglukosewerte über 110 mg/dl vor ist ein sofortiger Therapiebeginn mit Insulin erforderlich [12]. Der HbA_1c_ Wert ist für die Diagnose eines GDM ungeeignet, kann aber zur Verlaufskontrolle der Metabolik herangezogen werden und soll jedenfalls im Referenzbereich für Gesunde liegen (HbA_1c_ < 6,0 %). Die mütterlichen Blutzuckerprofile müssen auch während der Geburt im Zielbereich liegen (80–130 mg/dl), um neonatale Hypoglykämien und Anpassungsstörungen zu vermindern.

**Table 3:** 

Zeitpunkt	Kapilläres Vollblut (mg/dl)
Nüchtern (präprandial)	65–95
1 h postprandial	< 140
2 h postprandial	< 120

Zur weiteren Individualisierung der Therapie dient die fetale Biometrie, die zur Entscheidung, ob eine medikamentöse Therapie begonnen, intensiviert oder gelockert werden muss, herangezogen werden soll [12]. Der fetale Abdomenumfang korreliert mit dem fetalen Insulinspiegel. Liegt eine fetale asymmetrische Wachstumssteigerung vor und liegt die abdominelle Zirkumferenz über der 75. Perzentile des Gestationsalters sind strengere Therapieziele anzustreben. Hierbei sollen Nüchternglukosewerte bei kapillärer Messung < 90 mg/dl und 1 h postprandial < 130 mg/dl erreicht werden. Bei fötaler Wachstumsretardierung sind dementsprechend auch individuell angepasste, höhere mütterliche Glukosegrenzwerte zulässig bzw. ist der Beginn einer Insulintherapie zurückhaltend zu wählen [12].

Regelmäßige Kontrollen sollen individuell den Bedürfnissen der schwangeren Frauen entsprechend im Abstand von wenigen Tagen bis 3 Wochen erfolgen. Dabei ist anhand der Blutglukoseprofile eine Therapieanpassung (Insulindosis) je nach Erfordernis durchzuführen. Der Blutdruck und die Gewichtszunahme sollten kontrolliert und ein Harnbefund erhoben werden.

Auch telemedizinische Visiten können die Betreuungsqualität erhöhen und können vor allem bei Zugangsbeschränkungen aufgrund COVID 19 und zur Reduktion der Patientenzahlen in der Ambulanz als Social Distancing Maßnahme als Alternative überlegt werden. Telemedizinische Visiten bei GDM konnten in einer Metanalyse das Risiko für GDM assoziierte Geburts- und Schwangerschaftskomplikationen im Vergleich zur Standardbetreuung reduzieren [11, 33].

#### Insulin

Aufgrund der sehr gut dokumentieren Datenlage für Insulin sollte bevorzugt Insulin in der Schwangerschaft verwendet werden. Primär wird NPH-Insulin als Basisinsulin verwendet. Auch andere Langzeitinsuline (Glargin, Glargin U300 oder Detemir) können ohne Bedenken in der Schwangerschaft angewendet werden, jedoch zeigt eine Metaanalyse keine signifikanten Differenzen in Bezug auf mehrere maternale oder neonatale Parameter im Vergleich zu NPH-Insulin [34]. Im Vergleich NPH zu Glargin gibt es keine Unterschiede im Geburtsgewicht, sowie vergleichbares Risiko für neonatale Komplikationen und Malformationen. Ebenso sind die mütterlichen Outcomes Präeklampsie und Schwangerschaftshypertonie vergleichbar selten. Für Insulin Detemir ist das Risiko für LGA oder neonatale Hypoglykämie ebenso vergleichbar mit NPH Insulin [34]. Randomisiert kontrollierte Studien zu Insulin Degludec zeigen wie bereits zuvor publizierte Fallbeschreibungen keinen Hinweis auf maternale oder kindliche Komplikationen in der Schwangerschaft beziehungsweise vergleichbare Resultate wie bei anderen Langzeitinsulinen [35–38].

Schnellwirksame Insuline werden zur Korrektur postprandialer Spitzen angewendet. Vielfach finden Insulin Lispro oder Aspart Anwendung und sollten gegenüber Humaninsulin auch aufgrund der einfacheren Handhabung vorgezogen werden. Zu Glulisin liegen nur Vigilanzdaten in der Gravidität vor [39], die keine besonderen Auffälligkeiten in der Schwangerschaft zeigen, jedoch sollte es derzeit aufgrund der unzureichenden Datenlage nicht verwendet werden. Die Analoga konnten aber bisher nur teilweise Überlegenheit gegenüber Normalinsulin zeigen [34]. Der Vergleich von Aspart zu Humaninsulin zeigt keine Unterschiede bei Makrosomie oder Häufigkeit von Kaiserschnittgeburten. Lispro verglichen zu Humaninsulin war mit niedrigerer Inzidenz für Ikterus und weniger maternalen Hypoglykämien verbunden, andererseits wurden in der Gruppe mit Lispro höhere Inzidenzen für LGA und höheres Geburtsgewicht berichtet. Ultraschnell-wirksames Insulin Aspart (Fiasp) und ultrarapid Lispro (URLi, Lyumjev) sind in der Schwangerschaft zugelassen. Ultraschnellwirksame Insuline werden rascher resorbiert, sind daher schneller wirksam als bisherige Analoginsuline und werden zur Optimierung postprandialer Hyperglykämien angewendet.

#### Orale Antidiabetika

Der Sulfonylharnstoff Glibenclamid und das Biguanid Metformin werden in manchen Therapieempfehlungen (z. B. NICE, ADA Guidelines) als mögliche Alternativen oder zusätzlich zu Insulin in der Schwangerschaft genannt. Die Empfehlung wurde aber zuletzt aufgrund ungewisser Langzeitfolgen bei Nachkommen abgeschwächt [11, 12]. Metformin und Glibenclamid sind plazentagängig. Randomisierte kontrollierte Untersuchungen über den Einsatz von Glibenclamid und Metformin [40, 41] bei GDM zeigten keine wesentlichen Unterschiede zwischen der oralen Behandlung und einer Insulintherapie. Bei Verwendung eines dieser Präparate in der Schwangerschaft sollten die Patientinnen in die Therapieentscheidung miteinbezogen und aufgeklärt werden. Die Verabreichung von anderen oralen und subkutanen glukosesenkenden Medikamenten wie Alpha-Glukosidasehemmer, Glitazone, Glinide, GLP-1-Analoga, DPP-4- und SGLT-2-Hemmer wird in der Schwangerschaft nicht empfohlen. Es fehlt neben den Studiendaten zur sicheren Anwendung auch die Zulassung in der Schwangerschaft.

##### Metformin

An die Gabe von Metformin sollte insbesondere bei übergewichtigen insulinresistenten Frauen als Monotherapie oder in Kombination mit Insulin gedacht werden [26]. Unter Gabe von Metformin ab der 20. Schwangerschaftswoche wurde eine niedrigere Rate schwerer neonataler Hypoglykämien, jedoch eine höhere Frühgeburtenrate beobachtet [40]. Eine rezente Metaanalyse zeigt bei Frauen mit Metformintherapie verglichen zu einer Insulintherapie eine geringere maternale Gewichtszunahme in der Schwangerschaft, weniger Hypoglykämien bei Mutter und Kind sowie ein geringeres Geburtsgewicht [42]. Die Mütter in der Metformingruppe konnten bei der Nachuntersuchung postpartal eher ihr Ausgangsgewicht erreichen als insulinbehandelte Frauen; bezüglich des postpartalen Glukosetoleranzstatus bestanden keine Unterschiede [40]. Ein Grund für einen zögerlichen Einsatz von Metformin ist das Fehlen ausreichender Langzeitdaten zur kindlichen Entwicklung. Die MIG-TOFU Studie zeigte, dass Kinder aus der Metformintherapie-Gruppe in der Schwangerschaft erhöhte subkutane Fettmasse verglichen zur Insulingruppe aufwiesen – die Gesamtkörperfettmasse blieb jedoch vergleichbar [43]. Eine weitere Studie bei Nachkommen von Müttern, die bei PCOS 1700–2000 mg Metformin in der Schwangerschaft erhielten, konnte 4 Jahre nach Entbindung ein deutlich erhöhtes Risiko für Übergewicht und Adipositas im Vergleich zur Placebogruppe feststellen [44]. Bei Frauen mit PCOS und Metformintherapie zu Beginn der Schwangerschaft zur Ovulationsstimulierung wird eine Beendigung der Metformintherapie vor Ende des ersten Trimesters empfohlen [11].

##### Sulfonylharnstoff

Bei Ablehnung einer notwendigen Insulintherapie stellt eine Therapie mit Glibenclamid eine mögliche, wenn auch hierzulande äußerst selten genutzte Alternative zur Behandlung eines GDM dar. Eine Insulintherapie sollte aber jedenfalls präferiert werden, wenn der GDM bereits vor der 25. Schwangerschaftswoche besteht oder Glukosewerte über 110 mg/dl vorherrschen [26]. Vorteile von Metformin gegenüber Glibenclamid konnten in einer Metaanalyse gezeigt werden – unter Glibenclamidtherapie konnten höhere maternale Gewichtszunahme, sowie vermehrte Raten von fetaler Makrosomie und neonataler Hypoglykämie festgestellt werden [45]. Auch im Vergleich zu Insulin häufte sich unter Glibenclamid das Risiko für Makrosomien, neonatalen Hypoglykämien und höherem Geburtsgewicht [45]. Neugeborene aus Glibenclamid behandelten GDM-Schwangerschaften weisen zudem höheres Risiko für Geburtskomplikationen (Hypoglykämie, Atemnotsyndrom des Neugeborenen (RDS), Intensivstationsaufenthalte, LGA) auf [46]. In einer randomisiert kontrollierten Studie konnte ein höheres Risiko an kumulativ perinatalen Komplikationen (Makrosomie, Hypoglykämie, Hyperbilirubinämie) in der Glibenclamidgruppe im Vergleich zu Insulin festgestellt werden [47]. Zwar konnten keine erhöhten Makrosomieraten im Vergleich Glibenclamid zu Insulin beobachtet werden, jedoch waren die Hypoglykämieraten bei geringen Glibenclamiddosen von durchschnittlich 5,4 mg signifikant höher als unter Insulin. Aufgrund der Datenlage ist die primäre Verwendung von Insulin zur Behandlung von GDM klar zu favorisieren [48]. Eine Anwendung von Sulfonylharnstoffen in der Schwangerschaft wird nicht empfohlen.

### Geburtshilfliche Überwachung

Schwangere mit GDM sollten in einem Krankenhaus mit diabetologischer Erfahrung und angeschlossener Neonatologie entbunden werden. Empfohlen werden:

- Ein- bis dreiwöchentliche klinische Kontrollen
- bei Hyperglykämie in Frühschwangerschaft: Frühes Organscreening durch Ultraschall zum Ausschluss von Fehlbildungen (v. a. Herz, Niere)
- Ultraschall (Biometrie, Fruchtwasser, evtl. Doppler), Wachstumskurven (v. a. Wachstumszunahme des Abdomens = asymmetrische Wachstumszunahme; Polyhydramnion) beachten
- Achten auf erhöhtes Risiko zur Entwicklung einer Schwangerschaftshypertonie, Präeklampsie, Infektionen
- Idealen Geburtstermin und Geburtsmodus festlegen

Schwangere Frauen, die einen GDM im Laufe der Schwangerschaft entwickeln, zeigen eine herabgesetzte Insulinsensitivität bereits vor der Schwangerschaft [49, 50]. Dies könnte zu oxidativem Stress in der ersten Phase der Schwangerschaft führen und eine Ursache für kongenitale Defekte darstellen [51]. Um große anatomische Fehlbildung zu erkennen, ist für jede schwangere Frau eine Routine Ultraschall-Untersuchung zwischen der 18. und 22. Schwangerschaftswoche empfohlen [52].

Da die mütterliche Hyperglykämie einen direkten Einfluss auf die fetale Hyperglykämie, Hyperinsulinämie und, letztendlich, auf das fetale Wachstum hat [53], sind sonographische fetale Wachstumskontrollen alle 2–4 Wochen empfohlen [54]. Regelmäßig durchgeführte Ultraschallkontrollen führen zu einem besseren neonatalen Outcome und sollen zu einem nicht ultraschall-basierten Management präferiert werden [55]. Hierbei soll das erwartete fetale Gewicht (EFW) durch die Vermessung vom Kopfumfang (KU), Abdomenumfang (AU) und Femurlänge (FL) geschätzt werden [56]. Unter den geburtshilflichen Komplikationen eines GDMs erkennt man die fetale Makrosomie, welche bereits ab der 24. Schwangerschaftswoche sonographisch diagnostiziert werden kann, wenn der Abdomenumfang (AU) eine akzelerierte Wachstumstendenz aufweist [57]. Eine übermäßige Fruchtwassermenge (Polyhydramnion) wird als Hinweis einer diabetischen Fetopathie gesehen, wobei bis dato keine Referenzwerte festgelegt wurden [58]. Die Messung des fetalen subkutanen Fettgewebes könnte als Zusatzparameter für die Evaluation der diabetischen Fetopathie herangezogen werden, dies ist aber heute aufgrund der mäßigen Reproduzierbarkeit der Messwerte noch nicht Teil der Routine Untersuchungen bei GDM Schwangerschaften [59].

Eine Überschreitung des Geburtstermins sollte bei Schwangeren mit insulinpflichtigem GDM vermieden werden.

Ob zwischen Schwangerschaftswoche 38 + 0 und 40 + 0 eine Geburtseinleitung stattfinden soll, soll individuell entschieden werden. Dabei sollen der Insulinbedarf, die Ultraschallbefunde (Kindsgewicht, Doppler, Fruchtwasser), maternale Erkrankungen wie Präeklampsie und die vorausgegangenen Schwangerschaftsverläufe in die Entscheidung miteinbezogen werden [60–62]. Eine Einleitung wegen schlechter Blutzucker-Einstellung vor Schwangerschaftswoche 38 + 0 sollte wegen frühgeburtlichkeitsbedingter Morbidität vermieden werden. Vielmehr sollte eine pränatale Optimierung der Blutzucker-Werte erfolgen.

Es ist bekannt, dass das Risiko für eine Schulterdystokie ab einem Geburtsgewicht von 4250 g signifikant ansteigt [63]. Ab einem geschätzten Geburtsgewicht von 4500 g sollte deshalb bei einer Schwangeren mit GDM eine Sectio empfohlen werden. Bei einem Schätzgewicht von 4000–4499 g sollte eine differenzierte Aufklärung der Schwangeren über individuell erhöhtes Schulterdystokie-Risiko erfolgen, insbesondere bei ausgeprägter Kopf-Abdomen Differenz.

### Überwachung und Management des Neugeborenen (Abb. 3)

Ein Routinemonitoring ist für eine Hochrisikopopulation an Neugeborenen sinnvoll, zu denen Kinder aus diabetischen Schwangerschaften bzw. solche, die aus einem anderen Grund einem erhöhten Risiko für die Entwicklung einer Hypoglykämie ausgesetzt sind, zählen (z. B. dystrophe Neugeborene; LGA-Babys).

**Figure 3:**
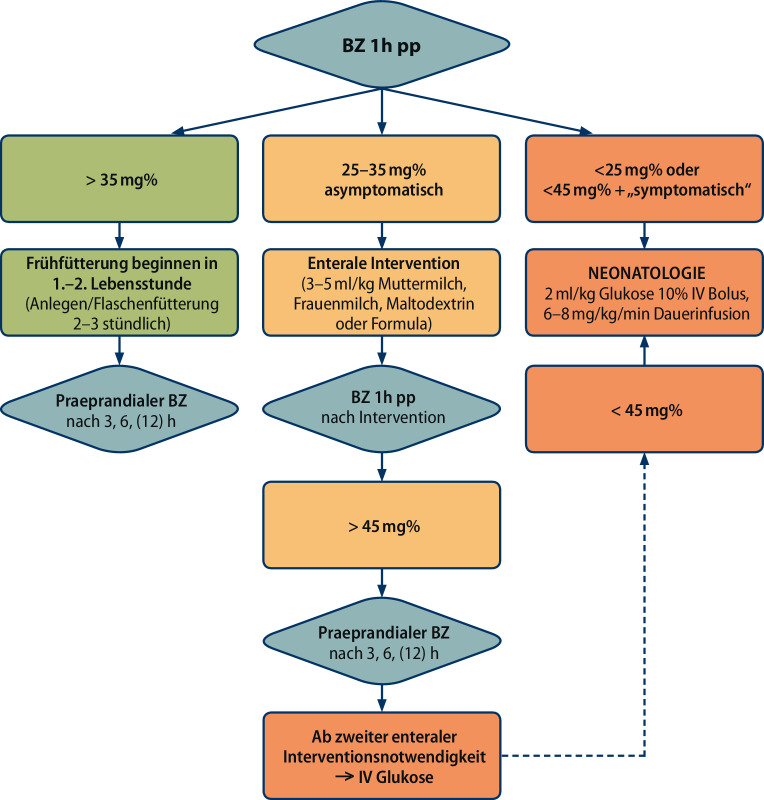

Generell zu vermeiden sind prolongierte und rezidivierende Hypoglykämien, da diese mit akuten systemischen und langfristigen neurologischen Konsequenzen einhergehen können [64].

#### Blutglukosebestimmungen nach Geburt

Erste Messung: In Abhängigkeit vom Risikofaktor soll bei zu erwartender Hypoglykämie 30–60 min nach Geburt (bei schlecht eingestelltem Gestationsdiabetes), ansonsten innerhalb der ersten zwei Lebensstunden gemessen werden. Der Einfachheit halber kann aber für die gesamte Population der Zeitpunkt 1 h nach Geburt festgelegt werden. Eine Ausnahme ist hier nur die Indikation schlecht eingestellter Schwangerschaftsdiabetes der Mutter – hier sollte die erste Messung bereits nach 30 min erfolgen.

Weitere Messungen: Zumindest zweimal vor den nächsten beiden Mahlzeiten (ca. nach 3 und 6 h, evtl. auch nach 12 h z. B. bei mütterlichem Diabetes, grenzwertigen Messungen). Ende der Messungen: Es sollen zumindest zwei normale präprandiale Glukosewerte hintereinander dokumentiert sein, um die Messungen beenden zu können.

Messung nach Intervention: Bei enteraler oder intravenöser Intervention aufgrund einer Hypoglykämie erfolgt eine Kontrolle 1 h nach Intervention.

Die Bestimmung der Blutglukose muss unmittelbar nach der Blutabnahme erfolgen. Bei Verwendung von Schnelltests (Glukometer) weisen diese im hypoglykämischen Bereich unter 45 mg/dl Glukose in Abhängigkeit vom Hersteller Ungenauigkeiten auf. Ein mit dieser Meßmethode ermittelter hypoglykämischer Wert soll durch eine laborchemische Bestimmung kontrolliert werden. Dies sollte aber zu keiner Verzögerung der Therapie führen.

#### Interventionsgrenzen und therapeutische Zielwerte

Aufgrund interindividueller Schwankungen gibt es keine absoluten Grenzwerte für die Behandlung der Hypoglykämie des Neugeborenen. Vorgeschlagen werden pragmatische „Interventionsgrenzen“ bei denen eine Intervention in Erwägung gezogen werden sollte (siehe Intervention: < 25 mg % intravenös, 25–35 mg % enteral).

Die „therapeutischen Zielwerte“ beinhalten einen Sicherheitsabstand.

#### Ernährung des Säuglings nach Geburt

Neugeborene sollen bereits innerhalb der ersten Lebensstunde angelegt werden, dies gilt besonders für Kinder aus diabetischer Schwangerschaft. Nahrung aus der Flasche (Anfangsmilch) soll nur angeboten werden, wenn Stillen nicht möglich/erwünscht ist bzw. als Intervention bei zu niedrigem Blutzucker (siehe „Intervention“) [65].

#### Intervention

Enteral: Nur bei asymptomatischer Hypoglykämie 25–35 mg % → Verabreichung von 10–20 ml (3–5 ml/kg) abgepumpter Muttermilch, Frauenmilch, Maltodextrinlösung 15 % oder Formulanahrung. Alternativ ist die Verwendung von buccal zu verabreichendem 40 %igen Dextrose-Gel möglich. Intravenös: Bei deutlicher Hypoglykämie < 25 mg %, symptomatischen Kindern < 45 mg % oder persistierender Hypoglykämie (falls die Kontrolle 1 h nach Intervention < 45 mg % ist, oder falls trotz zweimaliger enteraler Intervention weiter korrekturbedürftige präprandiale Blutzuckerwerte gemessen werden) → 2 ml/kg Glukose 10 % als iv Bolus, gefolgt von 6–8 mg/kg/min Glukose als kontinuierliche Infusion. Es wird eine schrittweise Reduktion der intravenösen Glukosezufuhr unter Beginn der enteralen Ernährung und präprandialen Blutzuckerkontrolle empfohlen.

Kinder von Frauen mit GDM haben ein höheres Risiko im späteren Leben übergewichtig zu werden und ein metabolisches Syndrom bis hin zu einem Diabetes zu entwickeln [66]. Deshalb sollte bei allen – und besonders bei makrosomen – Kindern auf eine normale Gewichtsentwicklung geachtet werden (s. „Nachbetreuung der Kinder“).

## Nachbetreuung der Mutter

Falls nach der Geburt normale Blutzuckerwerte erhoben werden (nüchtern < 100 mg/dl und unabhängig von Mahlzeiteneinnahme < 200 mg/dl) ist keine weitere definierte Ernährungstherapie oder Blutzuckerselbstmessung notwendig. Allerdings muss 4 bis 12 Wochen nach der Geburt eine Reklassifizierung der mütterlichen Glukosetoleranz mittels Standard-oGTT erfolgen. Bei pathologischem Befund müssen Therapieempfehlungen erfolgen (s. Leitlinien Diabetes mellitus – Definition, Klassifikation, Diagnose, Screening und Prävention, Diabetes-Therapie). Im Fall eines postpartal bestehenden Prädiabetes (gestörte Glukosetoleranz (2 h Wert 140–199 mg/dl) im oGTT oder erhöhter Nüchternglukose [100–125 mg/dl]) wird eine Lebensstiländerung mit Ernährungs- und Bewegungsberatung empfohlen. Eine Subanalyse des Diabetes Prevention Programs zeigte, dass bei vergleichbarer Ausgangslage bezüglich Glukosetoleranzstatus und Insulinresistenz Frauen mit GDM-Anamnese ein doppelt so hohes Risiko für die Progression zu einem manifesten Diabetes aufwiesen wie jene, die eine unauffällige Schwangerschaft hatten. Weiters profitierte diese Gruppe von einer Therapie mit Metformin besonders [67]. Dies wurde im 10 Jahres Follow up erneut bestätigt: Lebensstilmaßnahmen und Metformin konnten das Diabetesrisiko um 35–40 % verglichen zu Placebo verringern [68]. Eine Analyse des Wiener GDM Programms zeigte dass ein 2 h Blutzuckerwert im ersten oGTT postpartum über 140 mg/dl, ein HDL unter 50 mg/dl und ein Alter über 35 Jahre die wichtigsten unabhängigen Risikofaktoren für die Entwicklung eines manifesten Diabetes innerhalb von 10 Jahren darstellten [69]. Untersuchungen belegen nun auch für die seit einigen Jahren geltenden GDM Diagnoserichtlinie (basierend auf der HAPO Studie) ein mehr als 3‑fach höheres Risiko für eine Glukosestoffwechselstörung bei Frauen mit GDM im Vergleich zu Frauen mit normaler Glukosetoleranz nach 11 Jahren Follow up [70].

Entsprechend der Datenlage müssen alle Frauen mit GDM außerdem über ihr erhöhtes Risiko für die Entwicklung eines T2DM, eines GDM-Rezidivs (20–50 %) bei neuerlicher Schwangerschaft, ein erhöhtes kardiovaskuläres Risiko sowie über Möglichkeiten der Diabetesprävention informiert werden [70].

Bei unauffälligem Erstbefund sollen die Frauen alle zwei bis drei Jahre mittels oGTT oder zumindest mittels Messung der Nüchternglukose und des HbA_1c_ nachuntersucht werden.

Frauen mit Diabetes in der Schwangerschaft sollen, wenn immer es möglich ist, ihr Kind stillen, da protektive Effekte in Studien gezeigt werden konnten [26]. Bei einer Stilldauer von mehr als drei Monaten weisen stillende Mütter eine um bis zu zehn Jahre verzögerte Progression von GDM zu T2DM auf als nicht stillende Frauen [71].

Frauen nach GDM sollen reine Gestagen-Präparate insbesondere in der Stillzeit vermeiden, da sich dadurch das Risiko für die Manifestation eines T2DM erhöhen könnte [72]. Außer auf eine Glukosestoffwechselstörung soll auch auf weitere kardiovaskuläre Risikoparameter wie Dyslipidämie und Hypertonie untersucht werden, da Frauen nach GDM ein höheres kardiovaskuläres Risiko aufweisen [73, 74].

## Nachbetreuung der Kinder

Bei Nachkommen von GDM Schwangerschaften ist ein erhöhtes Risiko für Übergewicht/Adipositas und T2DM bekannt [70, 75]. Ein gesunder Lebensstil und regelmäßige Gewichtskontrollen sind zu empfehlen. Bei Hinweisen auf Hyperglykämie ist eine sofortige Abklärung empfohlen (siehe auch Leitlinie Diabetes mellitus – Definition, Klassifikation, Diagnose, Screening und Prävention). Ein T2DM-Screening sollte bei asymptomatischen Kindern und Jugendlichen bei Adipositas (BMI > 95. Perzentile, geschlechts- und altersadjustiert) oder ein Übergewicht (BMI > 85. Perzentile) und mütterlichem GDM in der Schwangerschaft des Kindes erfolgen [76].

## Evidenzlage

Gesichert ist, dass eine mütterliche Hyperglykämie im 1. Trimenon mit einem höheren Risiko für die Entwicklung einer diabetischen Embryopathie, im 2. und 3. Trimenon für die Entwicklung einer diabetischen Fetopathie mit erhöhter Morbidität und Mortalität assoziiert ist [77]. Die „Hyperglycemia and adverse pregnancy outcome (HAPO)“ Studie zeigte weiters, dass ein kontinuierlicher Zusammenhang zwischen der Höhe der mütterlichen Blutzuckerwerte im oGTT und den kindlichen Komplikationen besteht [17, 70, 78].

Während eine Ernährungstherapie alleine in Metaanalysen nicht eindeutig zu besseren fetalen Ergebnissen führt, ist die Verbesserung der postprandialen Blutglukosewerte unter Insulintherapie mit einer geringeren Morbidität verbunden. Studien konnten belegen, dass eine Behandlung (Ernährung und Bewegung, je nach Glukosewerten Insulin) des GDM das Risiko für schwere kindliche Komplikationen im Vergleich zu unbehandelten Frauen signifikant reduzieren konnte [3, 4].

Es konnte klar gezeigt worden, dass Frauen nach GDM ein besonders hohes Risiko für die Entwicklung eines T2DM haben [1, 6, 70], und auch ein höheres Risiko für die Entwicklung kardiovaskulärer Erkrankungen vorliegt [79, 80]. Lebensstiländerungen im Sinne der Diabetesprävention führen zu einer deutlichen Verringerung der Diabetesmanifestationsrate [67, 68]. In der Schwangerschaft war vor allem in Risikogruppen der Erfolg der bisherigen GDM Präventionsstudien bescheiden oder nicht vorhanden [22, 81]. Präzisionsmedizin-Ansätze könnten in der Zukunft zu größeren Erfolgen beitragen [82]. Kinder von Frauen mit GDM haben ein höheres Risiko selbst übergewichtig zu werden und Stoffwechselstörungen zu entwickeln [70, 78].

## Interessenkonflikt

A. Kautzky-Willer hat von folgenden Unternehmen, die teils auch fördernde Mitglieder der ÖDG sind, Forschungsunterstützungen und/oder Honorare erhalten: Astra Zeneca, Bristol-Myers Squibb, Glaxo Smith-Kline Pharma, Eli Lilly, Merck Sharp&Dohme, Novartis, Novo Nordisk, Roche, Sanofi-Aventis. Y. Winhofer hat von folgenden Unternehmen, die auch fördernde Mitglieder der ÖDG sind, Forschungsunterstützungen/Honorare erhalten: Sanofi Aventis, Eli Lilly, Astra Zeneca, Novartis. M. Lechleitner hat von folgenden Unternehmen, die auch fördernde Mitglieder der ÖDG sind, Honorare erhalten: Astra Zeneca, Bristol-Myers Squibb, Germania Pharmazeutika, GlaxoSmithKline Pharma, Eli Lilly, Medtronic, Merck Sharp&Dome, Novartis, Novo Nordisk, Pfizer, Sanofi-Aventis, Takeda. R. Weitgasser hat von folgenden Unternehmen, die teils auch fördernde Mitglieder der ÖDG sind, Forschungsunterstützungen und/oder Honorare erhalten: Abbott Diabetes Care, Amgen, Allergan, AstraZeneca, Boehringer-Ingelheim, Daiichi Sankyo, Eli Lilly, Merck Sharp & Dohme, Novo Nordisk, Roche Diabetes Care, Sanofi.. H. Kiss, V. Falcone, A. Berger und J. Harreiter geben an, dass kein Interessenkonflikt besteht.
